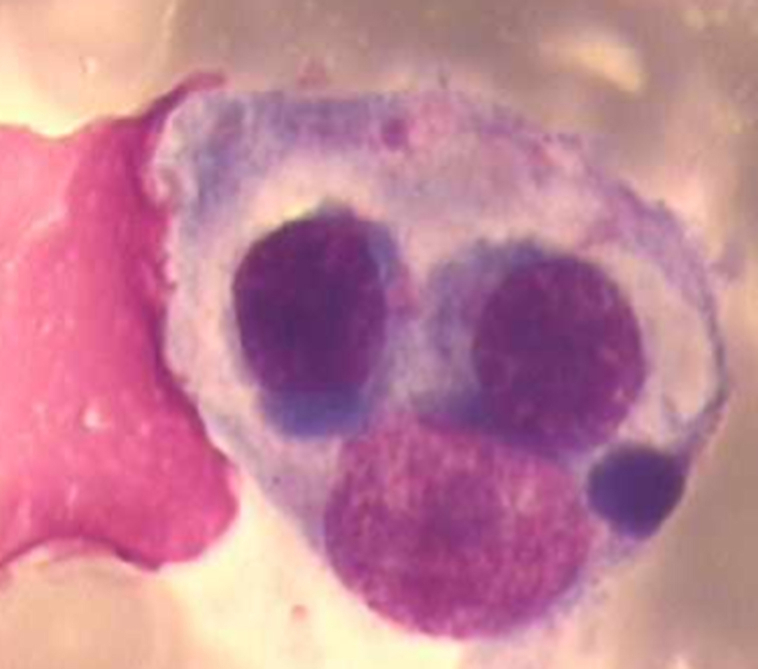# Septic arthritis caused by gout progressed to sepsis and hemophagocytic syndrome

**DOI:** 10.1016/j.heliyon.2024.e30583

**Published:** 2024-05-04

**Authors:** Wei Yang, Chun Yang

**Affiliations:** Wuming Hospital of Guangxi Medical University, Nanning, 530199, China; Department of Natural Resources of Guangxi Zhuang Autonomous Region, Nanning, 530022, China

**Keywords:** Gout, Septic arthritis, Sepsis, Hemophagocytic syndrome

## Abstract

Gouty stone ulcers inducing bloodstream infections leading to chest wall and mediastinal abscesses and progression to sepsis and hemophagocytic syndrome are extremely rare in clinical practice. Keep in mind the possibility of coexistence of gout and septic arthritis. Prompt and accurate diagnosis and treatment of hemophagocytic syndrome (HPS), a highly fatal disease with acquired immunoregulatory abnormalities and release of large amounts of inflammatory factors, are important to save the patient's life.

## Highlights

• Gout-induced septic arthritis progressing to sepsis and hemophagocytic syndrome.
• Septic arthritis due to gout is uncommon because of the positive role of medication in controlling hyperuricemia.
• The rare and fatal complications of sepsis and hemophagocytic syndrome in this patient illustrate the importance of aggressive treatment of gout.

## 1 Introduction

Due to the aggressive role of modern drug therapy in controlling hyperuricemia, ulcers caused by gout are rare, and the acidic environment of the wound itself makes infection extremely unlikely. As a result, it is easy to overlook this infectious factor, but when gout is complicated by sepsis and hemophagocytic syndrome, it is difficult to treat and has a high mortality rate. This case highlights a rare but important diagnostic and therapeutic challenge and illustrates the importance of aggressive treatment of gout and timely and accurate diagnosis and treatment of complications.

## 2 Case description

A 50-year-old Chinese man was referred to our hospital with a 10-day history of high grade fever. The patient had a 20-year history of gout, was not undergoing regular gout treatment, and was irregularly taking prednisone for pain at the onset of pain. The patient and family denied a past medical history of any other illnesses. On examination, he had a temperature of 40°, a blood pressure of 75/40 mmHg, a pulse rate of 136 beats/min, a respiratory rate of 35 breaths/min, and an oxygen saturation of 76 %, multiple gouty stone ulcers in the joints of the extremities and thick brownish fluid (Fig. 1,A). Laboratory tests showed hemoglobin 78 g/L (normal 120–160), platelets 29.0 × 10^9^/L (normal 100–300), white blood cell 1.63 × 10^9^/L (normal 3.5–9.5), procalcitonin 8.77 ng/mL (normal <0.05), fibrinogen 1.1 g/L (normal 2.0–4.0), alanine aminotransferase 454 U/L (normal 9–50), aspartate aminotransferase 613 U/L (normal 0–40), serum ferritin 3,100 μg/L (normal 15–200)and blood uric acid 563.5 ng/mL (normal 4.63–204). CT suggested a right chest wall abscess breaking and flowing into the chest cavity, mediastinal abscess, pneumonia (Fig. 1,B), and hepatosplenomegaly. Bone marrow examination showed hemophagocytic cells was increased. The thorax and mediastinum drained 500 mL and 200 mL of viscous tan fluid, respectively, and monosodium urate crystals were seen microscopically in the fluid flowing from the extremities; they both cultured the same antimicrobial spectrum of methicillin-resistant *Staphylococcus aureus* as the blood, and the same 16S ribosomal RNA.

**Fig. 1:**
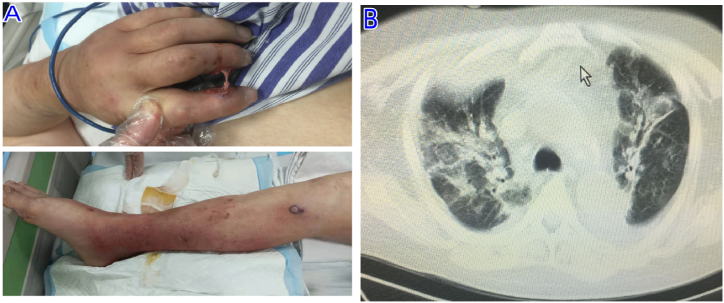
**(A)**Multiple gouty stony ulcers in the joints of the extremities and effusion of thick, brownish fluid. (**B)**The right chest wall abscess has broken down and flowed into the thoracic cavity, the mediastinum became wider and larger, showing aqueous hypodense shadow with inconsistent density (arrow).

Monosodium urate crystals (MSU) are the gold standard for the diagnosis of gout, and bacterial cultures of extremity joint secretions showed methicillin-resistant *Staphylococcus aureus* (MRSA), suggesting that gout coexists with septic arthritis. The diagnosis was gout-induced septic arthritis with infection through the bloodstream leading to chest wall and mediastinal abscesses, which progressed to sepsis, septic shock, and hemophagocytic syndrome (HPS) because the infection was not diagnosed and treated in a timely manner. Treatment was given with ventilator support, vancomycin and meropenem to strengthen anti-infection, and gammaglobulin to improve the body's immunity. Hydrocortisone shock therapy was administered for septic shock and HPS, and etoposide was combined to treat HPS. Septic joints are surgically irrigated and debrided, and blood purification, plasma exchange for acute kidney injury and removal of inflammatory factors, and febuxostat for control of blood uric acid levels are administered. During treatment, voriconazole antifungal therapy was also added depending on the condition and bacterial culture. The patient had been improving for a while and had been taken off the ventilator and blood purification. However, weekly blood bacterial cultures were consistently positive, and we also performed three echocardiograms that did not reveal that the patient had infective endocarditis. He eventually died 25 days after admission due to infection and multi-organ failure.

## 3 Discussion

This case highlights a rare but important diagnostic challenge. Due to the active role of modern drug therapy in controlling hyperuricemia, gout-induced ulcers are now uncommon and their wounds are less likely to become infected due to the acidic environment. Thus, the combination of gout and septic arthritis is rare [1]. Both cause warmth and swelling in the joints, making it easier to ignore the infection, especially in long-term chronic gout sufferers. Therefore, bacterial culture of gouty ulcers to clarify whether there is an infection is necessary [2]. And, because of the rarity of this condition, most clinicians can easily overlook it. A severe anti-inflammatory response occurs in sepsis, leading to apoptosis of immune cells. Prolonged irregular use of glucocorticoids leads to decreased immunity and septic shock further disrupts immune activation and induction of HPS.

HPS is also known as hemophagocytic lymphohistocytosis (HLH). HPS is a systemic inflammatory response syndrome caused by abnormalities in immune regulation, causing a series of clinical symptoms and signs characterized by fever, hepatosplenomegaly, pancytopenia, and the presence of hemophagocytosis in the bone marrow, liver, spleen, and lymph node tissues, and it is a rapidly progressing and highly fatal disease [3,4]. Infections, tumors, and autoimmune diseases are the most common triggers of HPS, with various pathogenic microbial infections triggering about 50.4 % of cases [5]. Of these, appropriate antibiotic and supportive therapy for patients with infection-related HPS, especially sepsis, is the most important tool [4]. Sepsis and HPS can present equally well with fever, decreased white blood cells, and elevated inflammatory markers including ferritin, which can lead to underdiagnosis and confusion between the two. While sepsis is treated with a combination of therapeutic measures focusing on organ function salvage, HPS may require hormone therapy in combination with etoposide, especially in the early stages when the addition of a short course of hormones and/or intravenous immunoglobulin to control inflammatory factors is beneficial. By impairing host immunity in the later stages of the disease, the hormone fails to achieve its intended purpose [4,5]. The mortality rate for adults with HPS in intensive care medicine has been reported to be as high as 57 % [4]. Therefore, early recognition of HPS and appropriate treatment are essential to improve the success rate of treatment. The patient was admitted to the hospital with deterioration of mental and pulmonary function already present, suggesting poor control of HPS or infection, indicating a lack of timely diagnosis and treatment. The patient had recurrent septic episodes, and we also performed three echocardiograms that failed to detect infective endocarditis, which is consistent with their report that HPS complicates the infection and makes it more difficult to treat [4].

## 4 Conclusion

This case reminds us that gout and septic arthritis may coexist, and its induced sepsis and HPS are extremely rare in clinical practice, and timely and accurate diagnosis and treatment are very important to save the patient's life. The main cause of the disease is due to severe infections induced by gouty stone ulcers, emphasizing the importance of aggressively controlling blood uric acid levels to prevent gouty stone rupture. Medical education is particularly important for patients with long-term chronic gout and poor medical compliance.

## Data availability statement

Relevant data are submitted with the submission of the manuscript, and the corresponding author can be contacted for additional requests.

The publicly available repository where the data is stored: https://www.lanzouw.com/iVfXG1w6gzef.

## CRediT authorship contribution statement

**Wei Yang:** Writing – review & editing, Writing – original draft, Visualization, Validation, Supervision, Software, Resources, Project administration, Methodology, Investigation, Funding acquisition, Formal analysis, Data curation, Conceptualization. **Chun Yang:** Visualization, Validation, Supervision, Software, Project administration, Methodology, Investigation, Data curation, Conceptualization.

## Declaration of competing interest

The authors declare that they have no known competing financial interests or personal relationships that could have appeared to influence the work reported in this paper.

## Figures and Tables

**figs1:**
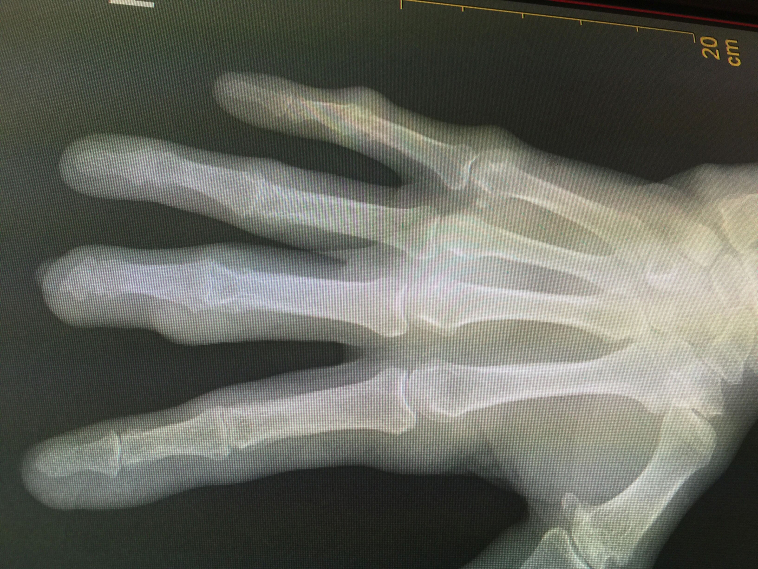


**figs2:**
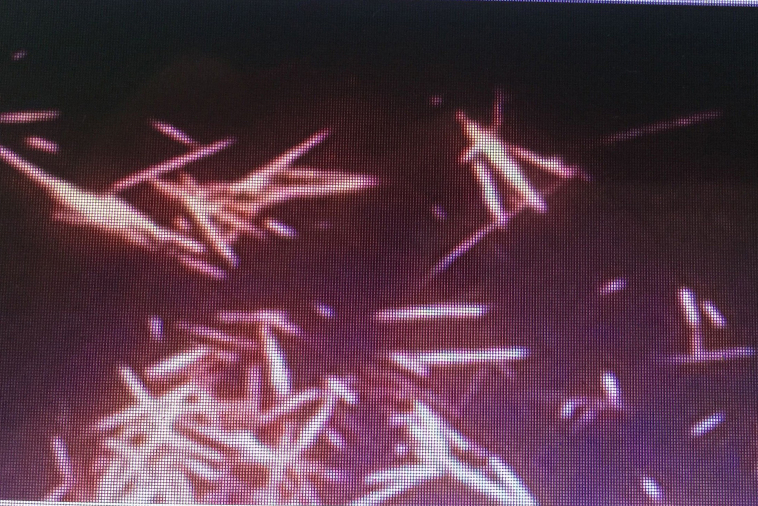


**figs3:**
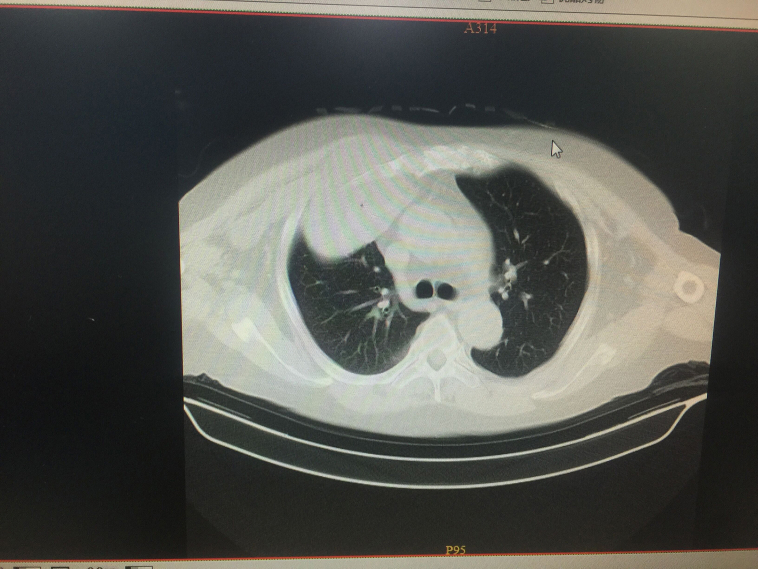


**figs4:**
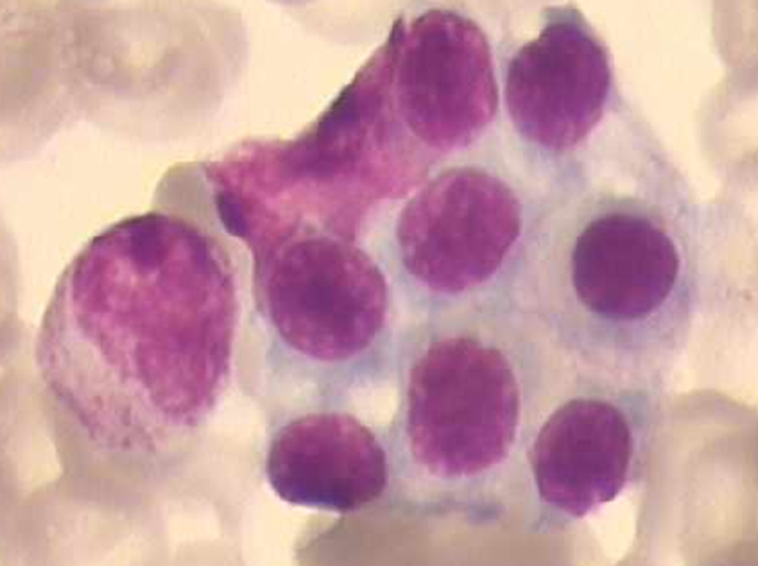


**figs5:**