# Supplementary material for: Septic arthritis caused by gout progressed to sepsis and hemophagocytic syndrome
Source: Heliyon. 2024 May 4;10(9):e30583. doi: 10.1016/j.heliyon.2024.e30583 (PMC11101825; doi:10.1016/j.heliyon.2024.e30583)
Supplement: Multimedia component 1 [file mmc1.pdf]

BMJ

同意书

适用于同意在英国医学杂志 (BMJ) 出版物中发表其图像和/或其信息的患者。

患者姓名： 王[illegible]

与患者的关系（如果患者未签署本同意书）： 女儿

*关于患者的照片、图像、文字或其他材料（材料）的描述。**应将这些材料的一份副本附在本同意书上：*** 胸部CT和骨髓像各一张

将包含材料的文章的暂定标题： Septic arthritis caused by gout progressed to sepsis and hemophagocytic syndrome

同意

我 王秋丽 *[用正楷书写全名]* 同意关于我/患者的材料出现在 BMJ 出版物中。

我确认我：（请在方框中打勾确认）

- ☑ *已看到关于我/患者的照片、图像、文字或其他材料。*
- ☑ *已阅读了提交给 BMJ 的这篇文章*
- ☑ *在法律上有权予以同意。*

我明白以下内容：

(1) 材料出版时不会附加我的/患者的姓名，但是我明白，不能保证材料完全匿名。可能某个地方的人——例如，照顾我/患者的某人或其亲戚——可能会认出我/患者。

(2) 材料可能会显示出或包含我的/患者的医疗状况或损伤以及我/患者现有的、已有的或将来会有的任何预后、治疗或手术的相关详细信息。

(3) 文章可能会发表在一份在全球范围内发行的杂志上。BMJ 出版物的读者主要为医生和其他医疗保健专业人员，但也有许多其他读者，包括学者、学生和记者。

(4) 包括材料在内的文章可能是一篇新闻稿的主题，也可能与社交媒体关联，和/或用于其他宣传活动中。文章一旦发表，就会放在 BMJ 网站上，可能还会在其他网站上提供。

(5) 文章的文字在发表前会对文体、语法和一致性进行编辑。

(6) 我/患者不会从文章发表中获得任何经济利益。

(7) 也可能在 BMJ 和/或其他出版商出版的其他出版物中全部或部分使用文章。这包括 BMJ 或其他出版商现在和将来以英语和翻译、印刷品、数字形式和其他任何形式出版的出版物。文章可能出现在英国和海外发行的地方版刊物或其他出版物中。

(8) 我可以在出版之前随时撤销我的同意，但是一旦文章已经准备好出版（"已付印"），则不可能撤销同意。

(9) BMJ 会按照法律的要求，安全和保密地保存同意书，直至不再有必要这样做为止。

请在方框中打勾确认如下内容:

☑ 我同意 BMJ 仅出于以后在必要时联系我的目的保存我的联系方式（包括在欧洲经济区 [EEA] 以外）。

☑ 当本同意与在 BMJ *病例报告*中的一篇文章相关时，我/患者有机会对该文章做出评论，我对该评论（如有）已在文章中得到反映感到满意。

签名: 王秋丽 *正楷书写姓名:* 王秋丽

地址: 中国广西南宁市武鸣区永宁路2号 *电子邮箱地址:* 19506258@qq.com

电话号码: +86 (152889671579)

如果代表患者签字，请给出患者不能自己予以同意的理由（例如，患者死亡，年龄在 18 岁以下，或患有认知或智力障碍）: 死亡 *日期:* 2023.7.10

☐ *如果您是为一个家庭或其他团体签名，请在方框中打勾，以确认该家庭或团体的所有相关成员均被告知*

如果患者为 7 岁或更大年龄的儿童，他们也必须确认他们同意这样做:

签名: ______ *正楷书写姓名:* ______

出生日期: ______ *日期:* ______

向患者或其代表（例如相应的作者或有权获得同意的其他人）**解释和给予同意书之人的详细信息。**

签名: Wei Yang *正楷书写姓名:* Wei Yang

职务: professor *地址:* Wuming Hospital of Guangxi Medical University, Naning China.

研究单位: Guangxi Medical Univeristy

电子邮箱地址: Yanggang20@126.com *电话号码:* 530199

日期: July 5, 2023.
